# Rapidity of the Progress of Cholera

**Published:** 1866-05

**Authors:** 


*Rapidity of the Progress of Cholera.*—A noteworthy fact in the history of epidemic cholera, is that it has never travelled with greater rapidity than *man can travel*. In India, says Prof. Clark, its march of progress was about 21 miles a week, sometimes less. In Europe it averaged from 80 to 100 miles a week, which is just about in the rate of increased facility and rapidity of travel in Europe as compared with India. In its crossing of seas, the Atlantic, for instance, it has travelled at a rate of about 300 or 400 miles a week, about the rapidity of the travel by vessels. During the late epidemic from Mecca to Alexandria, Ancona, Marseilles, Southampton, Paris, etc., it seems to have travelled with greater speed than formerly in Europe, owing, undoubtedly, to the increased facility and rapidity of intercourse by the modern means of locomotion.

Whatever general terrestrial or atmospheric causes of cholera there may be, its progress and spread seem dependent upon special and personal contingencies, and so long as there is such high probability that the cholera poison or the special miasm which forms one of its factors may be carried by persons or baggage, we hold that a humane but strict and uniform system of quarantine is a necessity, and we call upon Congress to bless the nation with a good quarantine code.—*Reporter.*

